# Uterine Epithelial Cells Specifically Induce Interferon-Stimulated Genes in Response to Polyinosinic-Polycytidylic Acid Independently of Estradiol

**DOI:** 10.1371/journal.pone.0035654

**Published:** 2012-04-25

**Authors:** Mickey V. Patel, Mimi Ghosh, John V. Fahey, Charles R. Wira

**Affiliations:** Department of Physiology and Neurobiology, Dartmouth Medical School, Lebanon, New Hampshire, United States of America; The University of Texas Health Science Center at San Antonio, United States of America

## Abstract

Interferon β (IFNβ) is an antiviral cytokine secreted in response to pathogenic exposure that creates a restrictive intracellular environment through the action of downstream interferon-stimulated genes (ISG). The objective of this study was to examine the expression of IFNβ and ISG in both human uterine epithelial cells (UEC) and the ECC-1 uterine epithelial cell line and determine if expression changes with TLR stimulation and hormone exposure. Stimulation of primary uterine epithelial cells and ECC-1 cells with the TLR3 agonist poly (I∶C) induced the mRNA expression of IFNβ, MxA, OAS2 and PKR. Other TLR agonists including imiquimod and CpG had no effect on either IFNβ or ISG expression. In contrast to ECC-1 cell responses which were slower, maximal IFNβ upregulation in UEC occurred 3 hours post-stimulation and preceded the ISG response which peaked approximately 12 hours after poly (I∶C) exposure. Unexpectedly, estradiol, either alone or prior to treatment with poly (I∶C), had no effect on IFNβ or ISG expression. Blockade of the IFN receptor abrogated the upregulation of MxA, OAS2 and PKR. Furthermore, neutralizing antibodies against IFNβ partially inhibited the upregulation of all three ISG. Estradiol, directly and in the presence of poly (I∶C) had no effect on IFNβ and ISG expression. These results indicate that uterine epithelial cells are important sentinels of the innate immune system and demonstrate that uterine epithelial cells are capable of mounting a rapid IFN-mediated antiviral response that is independent of estradiol and is therefore potentially sustained throughout the menstrual cycle to aid in the defense of the uterus against potential pathogens.

## Introduction

The female reproductive tract (FRT) is a unique mucosal site that must reconcile two competing functions: host defense versus reproduction. It is the primary site of infection by sexually transmitted diseases (STDs) including Herpes Simplex Virus (HSV), Human Immunodeficiency Virus (HIV), *Neisseria gonorrhoeae* and *Chlamydia trachomatis*, which cause morbidity and mortality in large numbers of women. The innate immune response in the FRT is a key component of host defense against these incoming pathogens about which much still remains to be elucidated.

Epithelial cells are essential for mucosal defense providing a physical and structural barrier against pathogen entry into the body. They are the first cells exposed to invading pathogens and thus, their responses could determine whether an infection occurs. Human uterine epithelial cells (UEC) express a full complement of pattern recognition receptors (PRR) including Toll-like receptors (TLR) 1–9, RIG-like receptors (RLR) and NOD-like receptors (NLR) [1], [2] that recognize unique and conserved pathogen-associated molecular patterns (PAMPs) allowing them to detect a plethora of infectious agents. TLRs 1, 2, 4, 5 and 6 recognize bacterial and fungal PAMPs [3]. TLR3 recognizes viral double-stranded RNA (dsRNA) while TLR7 and 8 recognize imiquidizalones such as imiquimod (IQ) and resiquimod as well as single-stranded RNA (ssRNA) [3]. TLR9 binds to unmethylated CpG DNA which is relatively rare in the vertebrate genome [3]. PAMP binding to a TLR activates multiple signaling pathways that allow a cell to generate a protective response. In previous studies we have demonstrated that UEC respond to bacterial exposure by secreting a panel of proinflammatory cytokines [1], [4].

Type I interferons (IFNs) are a family of cytokines that can rapidly induce a protective antiviral response and create a restrictive environment for pathogen survival [5]. Several subtypes of Type I IFN exist in humans including 13 isoforms of IFNα and single forms of IFNβ, IFNκ, IFNω and IFNε [6]. Type I IFNs are secreted into the external environment and signal in an autocrine and paracrine loop through a heterodimeric receptor complex consisting of IFN receptor 1 and IFN receptor 2 (IFNAR1 & 2) that activate a conserved JAK/STAT signaling pathway leading to the upregulation of hundreds of interferon-stimulated genes (ISG) [7]. ISG involved in inhibiting the viral lifecycle include Myxovirus A (MxA), 2′-5′ Oligoadenylate Synthetase (OAS) and Protein Kinase R (PKR). MxA, a high molecular weight cytoplasmic GTPase, is believed to exert its antiviral function by binding viral nucleocapsid proteins, forming aggregates and thus preventing mature virion formation and release [8]. OAS exists as 3 isoforms (OAS1-3) and catalyses the conversion of adenosine triphosphate (ATP) into long chains of 2′-5′ linked oligoadenylates [9], [10]. These molecules activate the endogenous ribonuclease, RNaseL, which digests viral RNA. Inactive PKR is present in the cytoplasm in monomeric form. Upon activation by viral RNA, it dimerizes and undergoes autophosphorlyation [9], [10]. In turn, activated PKR phosphorylates eukaryotic initiation factor 2 alpha (eIF2α) thus leading to a general suppression in protein synthesis [11]. Together, these three ISG represent a crucial mechanism behind Type I IFN-mediated defense – the ability to protect the cell by simultaneously inhibiting multiple stages of the viral lifecycle – and are capable of inhibiting a range of different viruses including Hepatitis C virus, HIV and HSV [12], [13], [14].

Every cell in the premenopausal FRT exists in an environment that is constantly exposed to varying levels of the sex hormones estradiol and progesterone. Previously, we have shown that estradiol regulates several aspects of the innate immune system in the FRT. In human UEC estradiol stimulates the production of antimicrobial compounds such as elafin and secretory leukocyte protease inhibitor (SLPI) that are essential in restricting viral and bacterial infections [15], [16]. However, estradiol can also be inhibitory. For example, it represses the TLR3-induced secretion of cytokines such as IL-6 and IL-8 in UEC [17]. Whether estradiol directly regulates the Type I IFN pathway, and thus alters intracellular innate immune protection has never been studied in UEC.

We examined the Type I IFN response of UEC to a panel of viral TLR agonists and demonstrated that UEC are exquisitely sensitive to dsRNA stimulation which transiently (over 24 hours) leads to a rapid intracellular antiviral response. Treatment with other viral TLR ligands did not induce a Type I IFN response. We examined the role of estradiol in modulating the IFN pathway and found that hormonal treatment does not alter the response profile of ISG in UEC. Overall, this study demonstrates the presence of a functional Type I IFN pathway in primary polarized UEC and the ECC-1 cell line that is responsive to the presence of dsRNA, is unaffected by the presence of estradiol and therefore provides a constant level of protection across the menstrual cycle.

## Materials and Methods

### Source of uterine tissue

Human uterine tissue was obtained immediately following surgery from premenopausal women who had undergone hysterectomies at Dartmouth-Hitchcock Medical Center (Lebanon, NH). Tissues used in this study were distal to the sites of pathology and were determined to be unaffected with disease upon inspection by a trained pathologist. All investigations involving human subjects were conducted according to the principles expressed in the Declaration of Helsinki and carried out with the approval from the Committee for the Protection of Human Subjects (CPHS), Dartmouth Hitchcock Medical Center, and with written informed consent obtained from the patients before surgery.

### Isolation of Uterine Epithelial Cells (UEC)

Epithelial cells were isolated as previously described [18]. Briefly, tissues were minced under sterile conditions into 1 to 2 mm fragments and subjected to enzymatic digestion using an enzyme mixture that contained final concentrations of 3.4 mg/ml pancreatin (Invitrogen Life Technologies, Carlsbad, CA), 0.1 mg/ml hyaluronidase (Worthington Biochemical, Lakewood, NJ), 1.6 mg/ml collagenase (Worthington Biochemical), and 2 mg/ml D-glucose, in 1× HBSS (Invitrogen). Enzymes were chosen to maximize digestion of the extracellular matrix while minimizing digestion of cell surface antigens. After enzymatic digestion for 2 hours at 37°C, cells were dispersed through a 250-µm mesh screen, washed, and resuspended in DMEM/F12 Complete medium. Complete medium was supplemented with 20 mM HEPES (Invitrogen), 2 mM L-glutamine (Invitrogen), 50 mg/ml primocin (Invivogen) and 10% heat-inactivated defined Fetal Bovine Serum (FBS) (ThermoScientific, Logan, UT).

Epithelial cell sheets were separated from stromal cells by filtration through a 20-µm nylon mesh filter (Small Parts, Miami Lakes, FL). Epithelial sheets were retained on the 20-µm filter, while stromal cells passed through. Epithelial sheets were recovered by rinsing and backwashing the filter with Complete medium. Epithelial sheets were collected, centrifuged at 500× *g* for 10 min, and analyzed for cell number and viability.

### UEC culture

To establish a cell culture system of polarized human UEC with both apical and basolateral compartments, human UEC were cultured in Falcon cell culture inserts coated with Human Extracellular Matrix (Becton Dickinson, Franklin Lakes, NJ) in 24-well culture plates (Fisher Scientific, Pittsburgh, PA). Apical and basolateral compartments had 300 and 850 µl of complete medium respectively. The medium was changed every 2 days.

### ECC-1 cell culture

ECC-1 cell line is a well-differentiated human UEC line that is responsive to sex hormones [19]. To establish a culture system of polarized human ECC-1 cells with both apical and basolateral compartments, the human UEC line ECC-1 (originally established by Dr Pondichery Satyaswaroop and kindly provided by George Olt, Penn State College of Medicine, Milton S Hershey Medical Center, PA) was cultured in uncoated Falcon cell culture inserts in 24-well culture dishes (Fisher Scientific). Apical and basolateral compartments had 300 and 850 µl of complete medium respectively. The medium was changed every 2 days.

### PBMC culture

Peripheral blood mononuclear cells (PBMC) were isolated from a blood cone and cultured in RPMI medium (Gibco) supplemented with 20 mM HEPES, 2 mM L-glutamine, 50 mg/ml primocin and 10% heat-inactivated defined FBS prior to TLR stimulation.

### TLR agonists, Interferon Neutralization and Receptor Blockade and Estradiol Stimulation

Polarized epithelial cells were apically stimulated with various TLR agonists at the following concentrations unless otherwise stated: poly (I∶C) (Invitrogen,) 25 µg/ml; imiquimod (Invivogen, San Diego, CA) 100 µM and CpG oligonucleotide (Invivogen) 1 µM.

Recombinant human IFNβ (PBL Interferon Source, Piscataway, NJ) was used to stimulate polarized UEC or ECC-1 cells for 24 hours. IFNβ neutralization experiments were conducted using a rabbit polyclonal anti-human IFNβ neutralizing antibody (αIFNβ) (R&D Systems, Minneapolis, MN). Interferon receptor blockade experiments were conducted using a mouse monoclonal anti-human interferon receptor 2 (IFNAR2) blocking antibody (R&D Systems)

For all hormone experiments, 17β-estradiol (Calbiochem, Gibbstown, NJ) was dissolved in 100% ethanol for an initial concentration of 1×10^−3^ M, evaporated to dryness and resuspended in Complete media containing charcoal dextran-stripped FBS to a concentration of 1×10^−5^ M. Further dilutions were made to achieve final working concentrations of estradiol ranging from 5×10^−8^ M to 5×10^−12^ M. As a control, an equivalent amount of ethanol without dissolved hormone was initially evaporated. In all cases, hormone was added to both the apical and basolateral compartments.

In all experiments with TLR agonists, IFN blockade and neutralization, or sex hormones, Complete medium containing 10% heat-inactivated FBS was replaced with Complete medium supplemented with 10% heat-inactivated charcoal/dextran-treated Stripped FBS (Gemini, West Sacramento, CA).

### Detection of IFNβ secretion

IFNβ secretion was analyzed by an enzyme-linked immunosorbent assay (R&D Systems). Briefly, cell culture conditioned media was recovered and centrifuged at 500 g for 5 minutes. The resulting supernatant was stored at −80°C until required.

### Measurement of transepithelial resistance (TER) and cell viability

As an indicator of tight junction formation of epithelial cell monolayers, TER was periodically assessed using an EVOM electrode and Voltohmmeter (World Precision Instruments, Sarasota, FL). All cell cultures had TER values greater than 1000 Ω/insert when experiments were performed. Cell viability was assessed using the CellTiter Cell Viability Assay (Promega, Madison, WI) as per the manufacturer's instructions.

### TaqMan real-time RT-PCR

Total RNA was isolated from cells using TRIzol Reagent according to the manufacturer's recommendations (Invitrogen) and purified on RNeasy columns (Qiagen, Valencia, CA) with on-column DNase digestion using the RNase-Free DNase set (Qiagen). 400 ng of total RNA was reverse-transcribed using the iScript cDNA synthesis kit (Bio-Rad) according to the manufacturer's recommendations. Relative mRNA expression levels of genes of interest were measured using the 5′ fluorogenic nuclease assay in real-time quantitative PCR using TaqMan chemistry on the ABI 7300 Prism real-time PCR instrument (Applied Biosystems, Carlsbad, CA). The IFN-β, OAS2, MxA, PKR, β-actin, progesterone receptor primer/MGB probe sets were obtained from Applied Biosystems assays-on-demand (ID nos. Hs00277188, Hs00942643, Hs00182073, Hs00169345, 4333762F and Hs01556702 respectively). PCR was conducted using the following cycle parameters: 95°C, 12 min for 1 cycle (95°C, 20 s; 60°C, 1 min), for 40 cycles. Analysis was conducted using the sequence detection software supplied with the ABI 7300. The software calculates the threshold cycle (C_t_) for each reaction and this was used to quantify the amount of starting template in the reaction. The C_t_ values for each set of duplicate reactions were averaged for all subsequent calculations. A difference in C_t_ values (ΔC_t_) was calculated for each gene by taking the mean C_t_ of each gene of interest and subtracting the mean C_t_ for the housekeeping gene β-actin for each cDNA sample. Assuming that each reaction functions at 100% PCR efficiency, a difference of one C_t_ represents a 2-fold difference. Relative expression levels were expressed as a fold-increase in mRNA expression and calculated using the formula 2^−ΔΔC^_t_.

## Results

### Effect of viral TLR agonists on cell viability and transepithelial resistance

**Figure 1:**
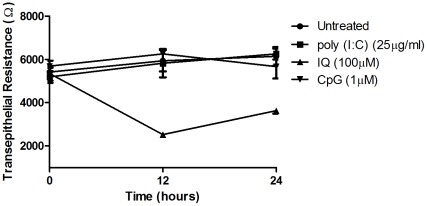
Imiquimod transiently decreases transepithelial resistance in ECC-1 cells. ECC-1 cells (*representative of three independent experiments*) were cultured on cell inserts until confluent and then apically stimulated with poly (I∶C) (TLR3), IQ (TLR7/8) and CpG (TLR9) at the concentrations shown above. TER measurements were taken at 12 and 24 hours post-stimulation. Results are shown as the mean +/− SEM.

Antiviral responses evoked by TLR stimulation can sometimes induce apoptosis as the cell seeks to limit potential sites of viral replication [20], [21], [22]. We sought to determine the effect of viral TLR stimulation on cell viability and found that TLR agonist treatment had no effect on epithelial cell viability compared to untreated samples (data not shown). We also investigated whether viral TLR agonists affect barrier function of epithelial cells by measuring changes in TER values of polarized ECC-1 cells. As seen in Figure 1, poly (I∶C) and CpG, when placed in the apical compartment, had no effect on TER at either 12 or 24 hours, relative to untreated controls. In contrast, IQ decreased TER by approximately 50% after 12 hours of exposure. At 24 hours post-exposure, in the continued presence of IQ, TER values had partially recovered but had not yet reached pre-treatment values.

### Viral TLR stimulation of uterine epithelial cells

**Figure 2:**
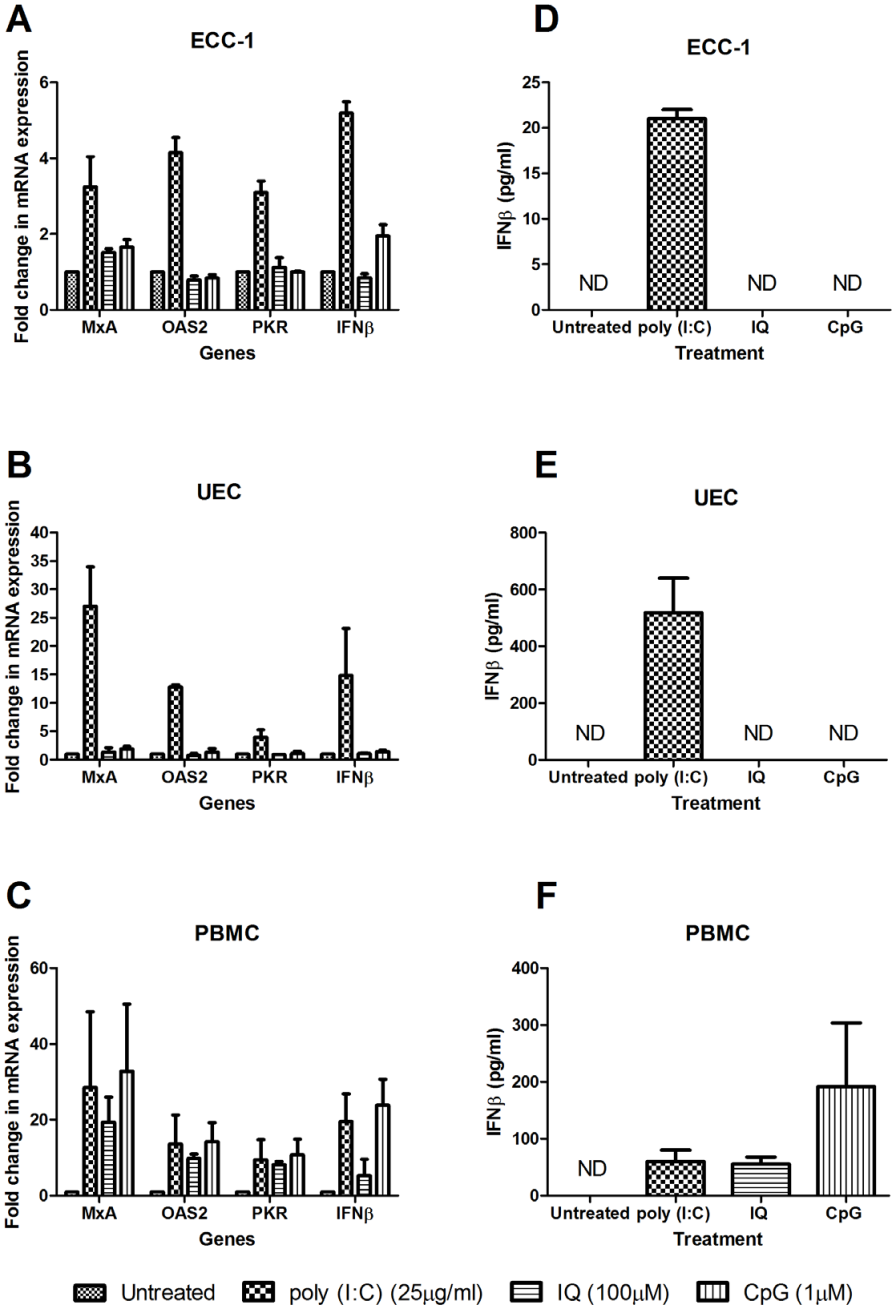
Epithelial cells induce IFNβ and ISG gene expression in response to poly (I∶C). ECC-1 (*n = 3*) (a & d), and primary human UEC (*n = 3*) (b & e) were cultured on cell inserts until confluent with a TER above 1000 ohms and then apically stimulated with poly (I∶C) (25 µg/ml), IQ (100 µM) and CpG (1 µM) for 24 hours. The same treatment was applied to primary human PMBC cultured in a 24-well plate (*n = 3*) (c & f). Cell culture conditioned media was recovered and analyzed by ELISA for IFNβ secretion (d, e & f). Concurrently total cellular mRNA was isolated and analyzed for changes in gene expression (a, b & c). mRNA is expressed as a fold change over untreated samples (assigned a value of 1). (ND = below detection limit of the ELISA assay).

Previously we have shown that ECC-1 cells and primary UEC express the full complement of human TLRs and that they respond to stimulation by upregulating proinflammatory cytokines and antimicrobials [1], [4]. However, the effect of viral TLR stimulation on Type I IFNs and ISG has not been fully determined. To test the hypothesis that viral TLR agonists regulate IFNβ and ISG expression, we apically treated confluent monolayers of ECC-1 cells or primary UEC for 24 hours with poly (I∶C), IQ and CpG before recovering and analyzing cell culture conditioned media and total cellular mRNA. The expression of MxA, OAS2, PKR and IFNβ was upregulated in response to poly (I∶C) (Figure 2a & b). In contrast, IQ and CpG added at concentrations known to have stimulatory effects on cytokine secretion [1], [23] did not induce IFNβ or ISG expression in either ECC-1 s or UEC (Figure 2a & b, d & e). Recognizing that one explanation for the lack of a response towards IQ and CpG could be due to inactive ligand, we repeated the experiments in PBMC. All three ligands upregulated MxA, OAS2 and PKR mRNA expression (Figure 2c). Furthermore, stimulation increased both IFNβ mRNA expression (Figure 2c) and secretion (Figure 2f) thus demonstrating that IQ and CpG were not defective and were able to induce gene expression in a different cell type.

### Poly (I∶C) stimulation of uterine epithelial cells

**Figure 3:**
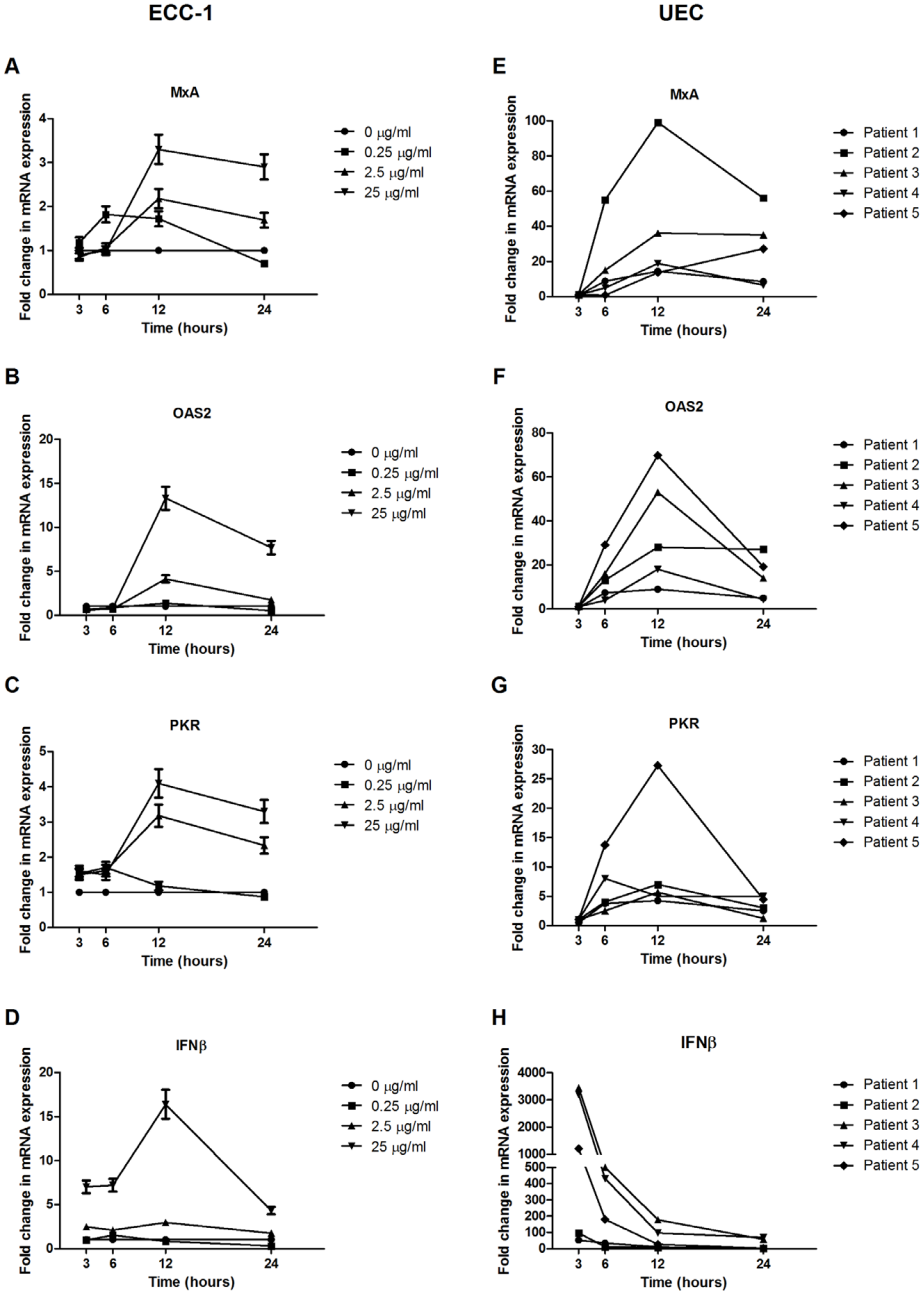
IFNβ transcription precedes the upregulation in ISG expression, with maximal ISG mRNA levels occurring approximately 12 hours after administration of poly (I∶C). ECC-1 (*n = 3*) (a–d) and primary human UEC (*n = 5*) (e–h) were cultured on cell inserts and then apically stimulated with poly (I∶C) for 3, 6, 12 and 24 hours. ECC-1 cells were stimulated with 0.25, 2.5 or 25 µg/ml of poly (I∶C) while UEC were stimulated with 25 µg/ml of poly (I∶C). Total cellular mRNA was recovered and analyzed for gene expression. mRNA is expressed as a fold change over untreated samples (assigned a value of 1).

To further elucidate the temporal response of IFNβ and the ISG, we exposed ECC-1 cells to multiple concentrations of poly (I∶C) for a range of time-points up to 24 hours. As shown in Figure 3, no increase in ISG was observed in ECC-1 cells at 3 and 6 hours post-stimulation. Maximum ISG expression occurred after 12 hours of treatment with mRNA levels of MxA, OAS2 and PKR increasing approximately 3-, 13- and 4-fold, respectively, beyond that seen in untreated controls (Figure 3a–c). An increase in IFNβ expression preceded that of the ISG with a 7-fold upregulation measured 3 hours after poly (I∶C) stimulation (Figure 3d). IFNβ expression peaked at 12 hours and then declined by 24 hours. Furthermore, poly (I∶C) demonstrated dose-dependent effects on gene expression with maximal levels of IFNβ and ISG reached with 25 µg/ml of poly (I∶C) and subsequently declining at 2.5 µg/ml and 0.25 µg/ml (Figure 3a–d).

In parallel studies with polarized human UEC, a similar pattern was observed. As seen in Figure 3e–h, ISG expression peaked at 12 hours post-stimulation when UEC were incubated in the presence of poly (I∶C) (25 µg/ml) placed in the apical compartment. However, unlike the ECC-1 cells, ISG levels were upregulated earlier at 6 hours post-stimulation. These studies further demonstrated that IFNβ peaked prior to the upregulation in ISG. In contrast to ECC-1 cells whose response was more gradual, primary UEC IFNβ expression was highest at 3 hours post-poly (I∶C) exposure (mean = 1600; range = 53–3444), and steadily declined towards 24 hours (mean = 30; range = 1.42–100).

### Inhibition of Type I IFN signaling

**Figure 4:**
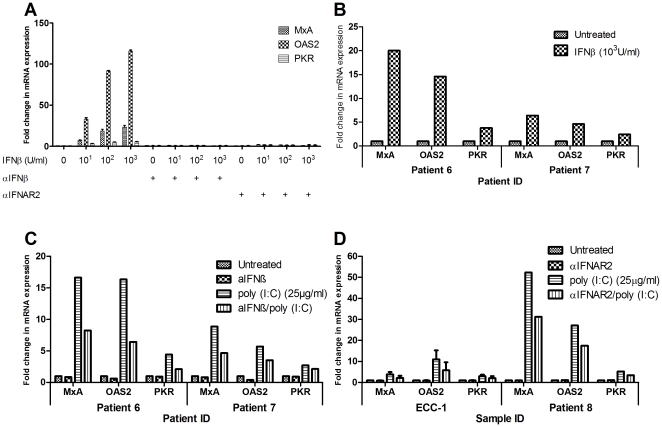
Inhibition of IFNβ signaling results in partial abrogation of poly (I∶C)-induced ISG upregulation. (a) ECC-1 cells (*n = 2*) were cultured on cell inserts until confluent and subsequently stimulated with multiple doses of recombinant human IFNβ (10^1^, 10^2^ & 10^3^ U/ml) for 24 hours (left panel) in the presence or absence of either neutralizing antibody for IFNβ (αIFNβ) (middle panel) or blocking antibody against interferon receptor 2 (αIFNAR2) (right panel). For the neutralization experiments αIFNβ and IFNβ were first mixed together in an eppendorf tube and then added to both compartments for 24 hours. For the receptor blockade experiments cells were pretreated apically and basolaterally with αIFNAR2 or IgG control (not shown) for 1 hour prior to apical stimulation with poly (I∶C) for a subsequent 24 hours during which αIFNAR2 was maintained in the cell culture media. (b) Primary human UEC from two individual patients were cultured on cell inserts until confluent and then stimulated with 10^3^ U/ml of recombinant IFNβ for 24 hours. (c) Concurrently, the UEC were treated with poly (I∶C) for 24 hours in the presence or absence of neutralizing antibody for IFNβ (αIFNβ). (d) ECC-1 cells (*n = 3*) or primary human UEC (Patient 8) (representative of 2 separate experiments) were cultured on cell inserts until confluent. Cells were pretreated with αIFNAR2 or IgG control (not shown) for 1 hour prior to apical stimulation with poly (I∶C) for a subsequent 24 hours during which αIFNAR2 was maintained in the cell culture media. Total cellular mRNA was recovered and analyzed for gene expression. mRNA is expressed as a fold change over untreated samples (assigned a value of 1).

The Type I IFN response has not been studied extensively in the human FRT epithelium despite it being a major site of STD transmission. To establish that polarized uterine cells are responsive to IFNβ, ECC-1 cells were incubated with a combination of recombinant human IFNβ (10^1^, 10^2^ & 10^3^ U/ml), IFNβ neutralizing antibody (αIFNβ) or IFNAR2 blocking antibody (αIFNAR2) for 24 hours prior to analysis of ISG (Figure 4a). IFNβ dose-dependently increased mRNA expression of MxA, OAS2 and PKR. OAS2 was most sensitive to the presence of IFNβ, increasing by approximately 30 (10^1^ U/ml) and 120- (10^3^ U/ml) fold over unstimulated levels, while MxA went up 6 and 20-fold respectively. In contrast, PKR was the least sensitive to IFNβ, increasing maximally by 6-fold at 10^3^ U/ml.

Addition of exogenous IFNβ (10^3^ U/ml) to UEC (Figure 4b) also induced MxA, OAS2 and PKR. However, the difference between the three genes was less stark with MxA and OAS2 both increasing between 5 to 20-fold (Figure 4b) over untreated levels. Similar to the ECC-1 cells, PKR appeared to be the least sensitive to IFNβ and was only upregulated by approximately 2 to 5-fold. As a part of these experiments, we concurrently treated the UEC with both poly (I∶C) and the αIFNβ antibody and discovered that expression of MxA, OAS2 and PKR were inhibited by approximately 20–40% (Figure 4c). To further elucidate the importance of IFNβ in the response to dsRNA, UEC and ECC-1 cells were pre-incubated with αIFNAR2 [24] for 1 hour and subsequently stimulated with poly (I∶C) for 24 hours. As shown in Figure 4d, antibody pre-incubation partially abrogated the upregulation of the three ISG in presence of poly (I∶C).

### Estradiol stimulation of uterine epithelial cells

**Figure 5:**
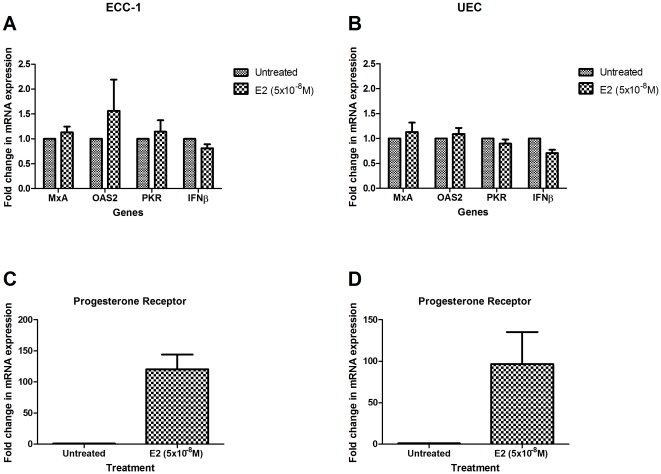
Estradiol has no effect on IFNβ and ISG transcript levels. ECC-1 (*n = 3*) (a & c) and primary human UEC (*n = 5*) (b & d) were stimulated apically and basolaterally by estradiol (5×10^−8^ M) for 24 hours. mRNA was recovered and analyzed for changes in gene expression. mRNA is expressed as a fold change over untreated samples (assigned a value of 1). Results are shown as the mean +/− SEM.

Epithelial cells in the upper FRT exist in an environment that is constantly exposed to levels of estradiol which vary with the stage of the menstrual cycle [25]. Previous studies from our laboratory have demonstrated that estradiol directly upregulates the production and secretion of protective antimicrobials including human β defensin-2 (HBD2) and SLPI, while inhibiting LPS and poly (I∶C)-induced secretion of MIF, IL-6 and IL-8 by primary UEC [16]. Recognition of the complex interactions between estradiol and UEC immune responses led us to investigate whether estradiol regulates IFNβ or ISG mRNA expression. ECC-1 and primary UEC were exposed to estradiol (5×10^−8^ M) apically and basolaterally. As seen in Figure 5, estradiol had no effect on IFNβ, MxA, OAS2 and PKR expression measured at 24 hours. In other experiments, when estradiol was added to the culture media for 3, 6, 12 and 48 hours (data not shown), no effects on IFNβ and ISG expression were seen in either ECC-1 or UEC. This lack of a response was not due to a defect in estradiol signaling pathways. Recognizing that estradiol stimulates the synthesis of progesterone receptor (PR) [26], we measured progesterone receptor mRNA levels and found increased expression at 24 hours in both ECC-1 and UEC (Figure 5c & d). Further, since ECC-1 and UEC TER values are known to decrease with estradiol treatment [16], we confirmed that hormone treatment decreased TER (data not shown) while having no effect on either ISG and IFNβ expression. Given that estradiol levels in blood vary with stage of the menstrual cycle, we extended these studies and carried out dose response experiments with ECC-1 cells at concentrations ranging from 5×10^−8^ M to 5×10^−12^ M over the same time (3–48 hr) periods. Irrespective of the dose of estradiol used, no change in IFNβ, MxA, and OAS2 or PKR expression was detected (data not shown).

### Estradiol and Poly (I∶C) co-stimulation of epithelial cells

**Figure 6:**
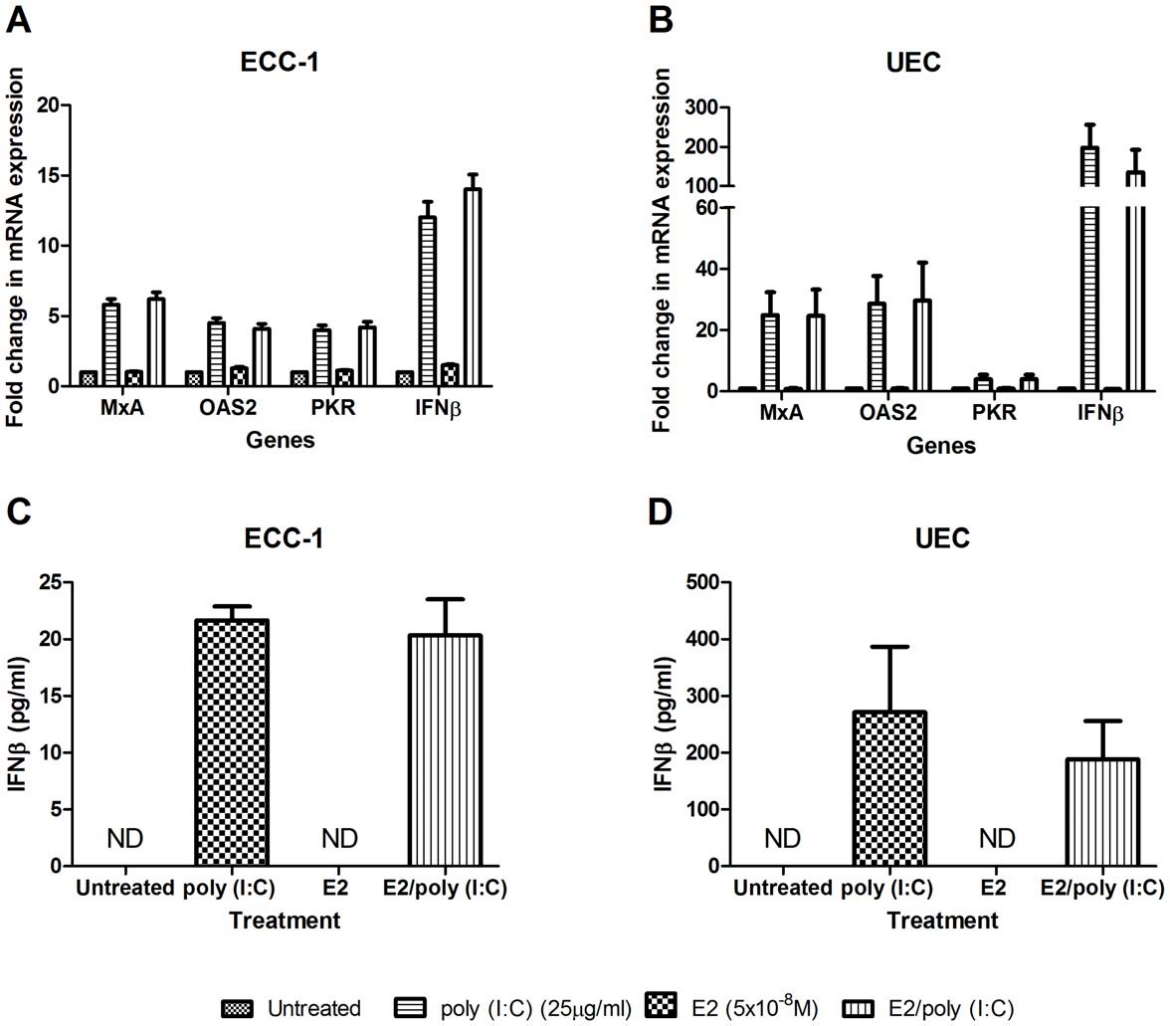
Estradiol has no effect on the poly (I∶C)-mediated increase in IFNβ, MxA, OAS2 and PKR. ECC-1 (*n = 3*) (a & c) and primary human UEC (*n = 3*) (b & d) were pretreated with estradiol (5×10^−8^ M) for 24 hours. Following media and hormone replenishment, poly (I∶C) (25 µg/ml) was added for 12 hours after which both cell culture conditioned media and mRNA were recovered and analyzed by ELISA (c & d) and RT-PCR (a & b) respectively. mRNA is expressed as a fold change over untreated samples (assigned a value of 1). Results are shown as the mean +/− SEM. ND = below the limit of detection of the ELISA assay.

Previous studies have shown that estradiol can regulate TLR-induced responses in the FRT [16], [17]. We therefore investigated whether estradiol would enhance or inhibit the upregulation of IFNβ and the ISG by poly (I∶C). To test this hypothesis, ECC-1 cells and primary UEC were pretreated apically and basolaterally with estradiol (5×10^−8^ M) for 24 hours followed by 12–24 hours incubation with poly (I∶C) during which estradiol was maintained in the culture media. As shown in Figure 6, estradiol had no effect on the poly (I∶C)-induced upregulation in IFNβ, MxA, OAS2 and PKR mRNA in either ECC-1 cells (6a) or primary UEC (6b) after 12 hours (not shown) or 24 hours of poly (I∶C) exposure. Furthermore, estradiol did not modulate the secretion of IFNβ in either cell type (Figure 6c & d). Additional experiments with ECC-1 cells using lower concentrations of hormone and extending the pretreatment period to 48 hours confirmed that estradiol had no effect on either IFNβ or ISG expression (data not shown).

## Discussion

These studies demonstrated that human UEC and ECC-1 upregulate IFNβ in response to poly (I∶C), a dsRNA viral mimic. When added to the culture media of polarized epithelial cells, IFNβ induced expression of MxA, OAS2 and PKR – a subset of ISG that protect against viral pathogens. Interestingly, inhibition of IFNβ action, either by blockade of IFNAR2 or by IFNβ neutralization, partially abrogated increases of MxA, OAS2 and PKR in the presence of poly (I∶C). The sex hormone estradiol had no effect on IFNβ or ISG expression, either alone or with poly (I∶C) stimulation. Overall, these studies indicate that ISG are present in uterine epithelial cells and under appropriate conditions, are up-regulated by IFNβ independently of estradiol.

Type I IFNs are secreted in response to pathogenic stimulation and rapidly induce an antiviral state in adjacent cells via their ubiquitously expressed cell-surface receptor complex consisting of IFNAR1 and 2 [5]. In turn, IFNAR signaling upregulates the expression of hundreds of proteins, many of which have been implicated in cellular defense from pathogen infection [5], [7]. MxA, OAS2 and PKR are three well characterized ISG whose expression we profiled after stimulation with synthetic agonists to TLR3, 7/8 and 9. We have demonstrated that the upregulation of IFNβ and the ISG (MxA, OAS2 and PKR) in UEC is specific to poly (I∶C), with no response to IQ or CpG at known stimulatory concentrations [2], [23]. Why TLR7/8 and TLR9 stimulation did not induce an IFN response in UEC is unknown. All three receptors are expressed and functional within UEC and ECC-1 cells [4], [27]. For example, TLR7/8 stimulation by loxoribine inhibits basolateral MCP-1 secretion in ECC-1 cells [1]. Primary cultured uterine cells upregulate the expression of CXCL8 in response to TLR7/8 and 9 agonists at similar doses to ours [2]. Elsewhere in the FRT, TLR7/8 and 9 induce IL-8 secretion in cervical epithelial cells [28], and MIP-1α in murine vaginal epithelial cells [29]. However, the unique sensitivity of UEC and ECC-1 to poly (I∶C) raises an intriguing question about their potential responses to pathogens they may encounter *in vivo*. HIV, for example, is recognized by TLR7 in specific subsets of immune cells. Could the inability of uterine epithelial cells to mount an effective Type I IFN response to TLR7/8 stimulation also imply they are insensitive to the presence of HIV and would this allow for increased viral transmission?

What confers epithelial sensitivity to dsRNA is unresolved but may involve TLR3 and potentially two additional PRRs known to recognize dsRNA: the cytosolic retinoic acid-inducible gene I (RIG-I) and melanoma differentiation-associated gene 5 (MDA5) which bind poly (I∶C) [30]. The presence of multiple receptors capable of recognizing dsRNA may dramatically increase cellular sensitivity to poly (I∶C). Both receptors are present in FRT epithelial cells (Wira and Ghosh, Unpublished observation). While the functionality of RIG-I and MDA5 in the uterine epithelium has never been studied, in lung epithelial cells RIG-I activation by influenza causes a Type I IFN-dependent antiviral response [31]. In contrast, TLR3 stimulation leads to a proinflammatory response. This could explain our past results where blockade of TLR3 signaling in UEC failed to inhibit IFNβ, MxA and OAS1 but not Human Beta Defensin 2 (HBD2) or TNFα [4]. Alternatively, the upregulation in IFNβ and the ISG may be due to the cumulative effect of all three PRRs signaling in synergy [32]. Further studies are needed to determine whether the PRRs act individually, or in combination, to mediate the effects of poly (I∶C) on IFNβ and ISG production. Overall, our past and present findings demonstrate that uterine ECC-1 epithelial cells and UEC recognize multiple TLR agonists but that the regulation of IFNβ and ISG expression is distinct from other induced immune responses in that their responsiveness is confined to dsRNA.

Unexpectedly, we found that when secretions from poly (I∶C)-stimulated cells were neutralized with an IFNβ-specific antibody, ISG upregulation in UEC was only partially abrogated (30–40%). Inadequate antibody blockade is unlikely since we used a concentration of antibody that abrogated IFNβ signaling in ECC-1 cells and was previously shown to completely block HSV-2-induced IFNβ signaling in UEC [23]. One explanation for our findings of a partial reduction is that, in addition to IFNβ, other Type I IFNs, such as IFNα or ε, are essential for the antiviral response in the human FRT. Recognizing that all Type I IFNs use the same receptor complex consisting of IFNAR1 and 2 to initiate signaling, we extended our studies and used a blocking antibody against IFNAR2 and achieved a partial reduction in ISG expression in the presence of poly (I∶C). Given the similar levels of ISG inhibition in both antibody experiments (30–40%), our results suggest that IFNβ is the dominant Type I IFN expressed by uterine epithelial cells – a feature observed in other cell types [33]. Alternatively, since we were unable to entirely block ISG upregulation, these studies suggest the existence of other IFN receptors capable of inducing ISG expression in epithelial cells. Signaling via the IL28Rα and IL10Rβ receptor complex by IL29 and IL28A/B has been demonstrated to upregulate MxA and OAS2 in human hepatic and intestinal epithelial cells [34], [35]. Whether these cytokines are expressed in the human endometrium is unknown, but they have been shown to function primarily at epithelial surfaces, induce a similar gene profile to Type I IFNs and are upregulated in response to poly (I∶C) in cell-specific manner [36], [37].

Epithelial cells are poised to respond to viral pathogens by rapidly upregulating IFNβ and subsequently, the ISG. Similar to findings elsewhere [38], in our system this response is promptly resolved and brought under control, and after 24 hours of poly (I∶C) exposure IFNβ and ISG mRNA responses were barely detectable. The similarity between the temporal dynamics and mechanisms of UEC and ECC-1 responses further indicates that ECC-1 cells are a valid model for studying the effects of viral stimulation on the IFNβ-mediated response in the upper FRT. Despite its protective properties, uncontrolled Type I IFN signaling is associated with increased inflammation and disease progression [39]. The tight control of IFN signaling observed here may be maintained via the action of several proteins including suppressors of cytokine signaling (SOCS) 1 and 3 as seen in human HepG2 cells and 293T cells [40], [41], [42].

The speed of the antiviral response probably allows uterine epithelial cells to confer protection against viral pathogens such as HIV and HSV that periodically enter the upper FRT after vaginal sexual intercourse. Type I IFNs are believed to have essential roles in restricting these pathogens [43], [44]. Indeed, loss of Type I IFN signaling is associated with increased HSV-2 acquisition in female mice [45]. Pretreatment of immune cells with IFN inhibits HIV replication [46], and both OAS2 and PKR are known to restrict HIV *in vitro* [47], [48]. In dendritic cells poly (I∶C) stimulation leads to increased secretion of Type I IFNs and subsequent upregulation of ISG that inhibit HIV replication in infected cells [44]. The exquisite sensitivity of uterine epithelial cells to dsRNA, a component of the HIV genome [49], may be essential in reducing male to female viral transmission. Given the importance of IFN-mediated protection for the host, both HSV2 and HIV encode viral proteins that actively subvert its ability to restrict their replication. The HSV-2 virion host shut-off (vhs) protein inhibits dsRNA-induced antiviral signaling in human vaginal epithelial cells by interfering with TLR3/MDA5 recognition and suppressing IFNβ secretion [50]. In a similar fashion, the HIV protein TAT induces SOCS3 expression to suppress IFNβ signaling and thus increase HIV replication in macrophages [51]. The ISG themselves are also targets of HIV, and both PKR and OAS2 can be subverted by the TAR element on HIV RNA and RNaseL inhibitor respectively [52], [53]. However, while the preponderance of evidence suggests that ISG are protective for the host cell, some studies have shown that ISG upregulation can predispose a cell to increased infection [14]. For example, HSV-1 induces the expression of a unique isoform of MxA that increases viral replication in human fibroblasts [54]. Therefore, it is important to appreciate the possibility that ISG upregulation may not always increase protection.

Previous studies by us demonstrated that estradiol and progesterone regulate a spectrum of uterine epithelial cell immune functions, including tight junction formation, cytokine and antimicrobial secretion, as well as antigen presentation [25]. For example, estradiol increases antimicrobial (SLPI and elafin) expression in the upper FRT at a time when other immune parameters are suppressed to optimize conditions for successful pregnancy [16]. The present studies build on this foundation in several ways. Estradiol had no effect on constitutive IFNβ, MxA, OAS2 and PKR mRNA expression. These findings suggest that Type I IFN and ISG expression in epithelial cells is insensitive to the presence of sex hormones and thus provides a constant level of protection across the menstrual cycle. In other studies, estradiol upregulated mRNA levels of ISG20/HEM45 in human UP1, MDA-L3 and MCF-7 tumor cell lines [55]. Our lack of an estradiol-induced response is in stark contrast to other aspects of the innate immune system in the FRT where we have demonstrated that estradiol has a significant regulatory role [16], [56]. To the best of our knowledge, our findings are the first to demonstrate that physiological concentrations of estradiol, which are biologically active in our cells in that they induce the expression of progesterone receptor (Figure 5), have no effect on Type I IFN signaling in uterine epithelial cells.

The present studies demonstrated that pretreatment of epithelial cells with estradiol had no effect on poly (I∶C)-induced IFNβ and ISG expression. These findings are in contrast to our previously published findings that cytokine and chemokine secretion in response to TLR agonists are dampened when epithelial cells are pretreated with estradiol [16]. For example, whereas estradiol had no effect on constitutive secretion of proinflammatory cytokines and chemokines by uterine epithelial cells, it inhibited LPS and poly (I∶C)-induced secretion of MIF, IL-6 and IL-8. Estradiol also reversed the stimulatory effects of IL-1α on mRNA expression of TNFα, IL-8 and NFκB [17]. Overall, the present study indicates that the production of IFNβ and ISG by UEC are selectively unresponsive to estradiol. Further, it suggests that the presence of IFNβ and ISG in epithelial cells which line the uterine cavity have evolved immunologically to be sensitive to viral infections throughout the menstrual cycle to enhance the chances of procreation. In contrast to our findings with epithelial cells, estradiol pretreatment of human dendritic cells inhibits the upregulation of IFNβ and MxA in response to Newcastle's Disease Virus (NDV) [57] without affecting IFNβ-induced RIG-I, MxA, IP10 or STAT1 expression [57]. That protection against viral infection occurs without accompanying inflammation is essential for successful fertilization and implantation. Further studies are needed to more fully understand the dynamic balance that exists between innate intracellular protection and reproductive function [58].

In conclusion, we demonstrated the presence of a robust IFNβ-mediated response to poly (I∶C) which is unaffected by estradiol in both human UEC and ECC-1 cells. The lack of an estradiol effect suggests a degree of protection that is unaffected by hormonal flux across the menstrual cycle thus indicating the importance of maintaining a constant level of Type I IFN-mediated innate immune protection.
